# Telehealth‘s future in Australian primary health care: a qualitative study exploring lessons learnt from the COVID-19 pandemic

**DOI:** 10.3399/BJGPO.2022.0117

**Published:** 2023-03-22

**Authors:** Christine Ashley, Anna Williams, Sarah Dennis, Susan McInnes, Nicholas A Zwar, Mark Morgan, Elizabeth Halcomb

**Affiliations:** 1 School of Nursing, University of Wollongong, Wollongong, NSW, Australia; 2 School of Nursing and Midwifery, Western Sydney University, Sydney, Australia; 3 School of Nursing and Midwifery, University of Notre Dame Australia, Sydney, Australia; 4 Sydney School of Health Sciences, The University of Sydney, Sydney, Australia; 5 Faculty of Health Sciences and Medicine, Bond University, Robina, Australia

**Keywords:** telemedicine, primary health care, sustainability, qualitative research

## Abstract

**Background:**

During the COVID-19 pandemic, telehealth emerged as a means of safely providing primary healthcare (PHC) consultations. In Australia, changes to telehealth funding led to the reconsideration of the role of telehealth in the ongoing provision of PHC services.

**Aim:**

To investigate GPs’, registered nurses‘ (RNs), nurse practitioners‘ (NPs), and allied health (AH) clinicians perceptions of the sustainability of telehealth in PHC post-pandemic.

**Design & setting:**

Semi-structured interviews were undertaken with 33 purposively selected clinicians, including GPs (*n* = 13), RNs (*n* = 5), NPs (*n* = 9), and AH clinicians (*n* = 6) working in PHC settings across Australia. Participants were drawn from responders to a national survey of PHC providers (*n* = 217).

**Method:**

The thematic analysis approach reported by Braun and Clarke was used to analyse the interview data.

**Results:**

Data analysis revealed that the perception of providers was represented by the following two themes: lessons learnt; and the sustainability of telehealth. Lessons learnt included the need for rapid adaptation to telehealth, use of technology, and the pandemic being a catalyst for long-term change. The sustainability of telehealth in PHC comprised four subthemes around challenges: the funding model, maintaining patient and provider safety, hybrid service models, and access to support.

**Conclusion:**

Providers required resilience and flexibility to adapt to telehealth. Funding models must reward providers from an outcome focus, rather than placing limits on telehealth’s use. Hybrid approaches to service delivery will best meet the needs of the community but must be accompanied by support and education for PHC professionals.

## How this fits in

Telehealth services had been underutilised in Australia before the COVID-19 pandemic owing largely to lack of infrastructure and funding. Rapid adoption by PHC since 2020 has identified its many benefits but has also highlighted limitations in current funding models to sustain its use in the long term. While a hybrid model of care delivery is the preferred approach, this must be accompanied by well-educated and upskilled health professionals to ensure optimal outcomes for patients.

## Introduction

Telehealth services have been funded in Australia since 2006 predominately to provide access to medical specialists (for example, cardiologists and respiratory physicians) and mental health consultations for people living in rural and remote areas.^1–3^ However, during the COVID-19 pandemic, strict isolation rules and the need to reduce face-to-face contact resulted in the widespread and rapid adoption of primary care telehealth services.^3,4^

Telehealth is a broad term referring to the use of information and communications technology to share audio, images, or data between a consumer and a health professional for the purposes of health assessment, diagnosis, and/or intervention.^5,6^ There is evidence that telehealth is largely accepted by communities^5,6^ and health professionals,^7,8^ and can facilitate high-quality, cost-effective health care resulting in positive health outcomes.^5^ Telehealth services are delivered in different modes including video, online platforms (for example, web and mobile apps), telephone, and via email.^6^ Advances in technology have enabled the increasing use of interactive and monitoring devices to record and transmit data directly from patients to health professionals.^5^

An integral component of the Australian national PHC response to COVID-19 was the introduction of additional Medicare Benefits Schedule (MBS) funding for telehealth services. This resulted in an exponential increase in the uptake of PHC telehealth consultations, with approximately 34% of GP consultations being conducted by telehealth,^2,9^ and recognition that 85% of patients were potentially manageable by telehealth.^9^

In Australia, a key barrier to the uptake of telehealth has been the limited access that RNs and NPs have had to MBS funding.^1^ Additionally, AH clinicians were limited to providing telehealth services to patients who held private health insurance or to those willing to pay for telehealth consultations.^8^ While initially the strict funding criteria that precluded nurses from accessing telehealth MBS items during the COVID-19 pandemic resulted in PHC nursing job losses,^10^ the broadening of criteria for reimbursement for telehealth consultations during 2020–2021 enabled RNs, NPs, and AH clinicians to provide funded telehealth services. This article seeks to inform post-pandemic policy about the factors that will influence the future viability of telehealth in PHC from the perceptions of health professionals.

## Method

A qualitative descriptive study^11^ was undertaken as part of a two-phase mixed-methods study exploring PHC professionals’ experiences of using telehealth during the COVID-19 pandemic. Phase one was a national survey of GPs, RNs, NPs, and AH clinicians working in PHC.^12^ Survey responders provided contact details if they were interested in taking part in subsequent interviews. Phase two consisted of interviews with purposively selected survey responders.

### Recruitment

Of the 217 survey responders, 70 indicated a willingness to be interviewed. These responders were stratified according to their profession, employment setting, geographic location, age, and sex. People were then purposively selected and contacted by one of three researchers, provided with study information, and invited to participate. Participants were recruited until data saturation was achieved, that is, the interviewers agreed that no new information was forthcoming.

### Data collection

As participants were geographically dispersed, interviews were conducted by telephone (*n* = 29) and videoconference (*n* = 4). Semi-structured questions and prompts were developed drawing on the literature and initial survey findings. Participants were invited to provide their reflections of using telehealth, lessons learnt, challenges experienced, and their views on future telehealth use. They were also asked to comment on their perceptions of patients’ experiences of telehealth during COVID-19. This study focuses on the future viability of telehealth.

After six interviews were conducted, the interview schedule was reviewed, and minor modifications made to ensure clarity and consistency. All interviews were digitally recorded, except one where the participant declined audiorecording. Field notes were taken by all interviewers. Interview duration ranged from 16–76 minutes (mean 36.5 minutes).

### Data analysis

Audiorecordings were professionally transcribed verbatim. Each transcript was checked against the recording and de-identified before being uploaded into NVivo (version 12). Analysis was undertaken using the approach reported by Braun and Clarke.^13^ Inductive analysis, where coding of data takes place without attempting to ‘fit in’ to any pre-conceived framework, resulted in two themes. Discussions between authors occurred to reach consensus on codes and themes. Supplementary Box S1 provides an overview of the strategies used to demonstrate trustworthiness.

## Results

Thirty-three interviews were conducted between July and August 2021. Participants included GPs (*n* = 13), RNs (*n* = 5), NPs (*n* = 9), and AH clinicians (*n* = 6). The AH clinicians comprised speech pathologists, physiotherapists, psychologists, and exercise physiologists. The perceptions of providers regarding the sustainability of telehealth was represented by two themes; lessons learnt, and sustainability of telehealth.

### Lessons learnt

Participants described lessons learnt in terms of the following three subthemes: 1) need to rapidly adapt; 2) use of technology; and 3) catalyst for long-term change.

#### Need to rapidly adapt

Many participants described having to quickly adapt to using telehealth and to rapidly adjust their clinical practice owing to the pandemic. Participants commonly commented on needing to be resilient to change and flexible in their approaches to service delivery:

*‘I think we’ve learnt how flexible and adaptable this workforce is. And I actually don’t think it’s been a huge problem, I think GPs have picked this up really fast … I think what they’ve learnt is, you know, you throw a pandemic at general practice and look at us ... we’ve done telehealth, we’ve done whatever it takes to get our patients seen.’* (GP12)

For some, the use of telehealth drew on their personal resilience and tested their capacity to adapt:

*‘There’s a lot of personal resilience, growth kinds of things for both myself and for my clients that just because you haven’t done something before doesn’t mean you can’t.’* (RN4)

*‘A bit more flexible with technology … I survived much better than I expected, so it’s really been a nice little — I know this sounds awfully stuck up, but quite a nice little pat on the back.’* (AH clinician5)

As well as the need to adapt as clinicians, as small business owners and employers, GP participants acknowledged that ongoing telehealth service delivery requires providers to regularly review and adapt to the changing telehealth funding guidelines. For many, this created additional workforce pressures at a time when resources were already stretched:

*‘So the MBS has changed — and you have to really keep up with that quite — and then, you know, I’ve got a practice with 25 doctors and I’m the medical coordinator, so basically to keep the business running everyone has to know what item number to use and what are the rules of engagement, basically.’* (GP3)

*‘We’re finding the guidelines we followed yesterday can’t be followed today and the ones we follow the day after — you know, we’re all trying to get ourselves up to speed on changes that are occurring almost overnight.‘* (GP12)

#### Use of technology

Regarding the use of technology, one RN described the following:

*‘... the majority of the consultations are by telephone … generally it’s just easier for everybody. There really isn’t much benefit, in my mind, to doing a video consultation in my type of work, and if I felt there was a specific need to see somebody, perhaps for the training of a technical device … then I would organise that. But I just find telephone does the job most of the time.’* (RN10)

To undertake successful video consultations:

*‘... you’ve got to have good systems, you’ve got to have good technology and you’ve got to do a lot of educating for it to go well.‘* (GP6)

However, others felt video consultations could be simplified:

*‘I think there’s a bit of a concern, or a perception of it’s quite a complex procedure to set up a video whereas with current technology it’s very straightforward. You can make it complex, but you only need basically a couple of bits and pieces and it can work.‘* (GP10)

Some participants also noted that successful telehealth reflected the broader community adjusting to a digital world:

*‘I think skills and confidence in using telehealth have improved, and from a client perspective, I would say that COVID outbreaks are higher,* [so] *people are more open to the idea of telehealth.’* (AH clinician1)

*‘... a lot of my patients struggle at first to be seen on the screen ... I always open Zoom with my cat on my shoulder, and then they talk to my cat and then they show me their pets and then we’d all be fine ... ‘* (GP12)

#### Catalyst for long-term change

Reflecting on the challenges created by the rapid adoption of telehealth, participants were largely enthusiastic that telehealth was now widely available, and that quality of care had not been compromised. It was considered important that providers understand telehealth systems, embrace change, and focus on the outcomes achieved:

*‘... general practice has changed forever now. This is what general practice is, it’s information management, it’s the software, it’s telehealth. You’ve got to really understand this stuff going forward, I suppose ... this is a whole massive change in 12 months and you’ve got to bite the bullet. You’ve got to say it’s general practice and really embrace the change.’* (GP3)

*‘I think we’ve learnt that it’s something not to be afraid of. Like, I’ve known for years and years and years we’ve campaigned for it, particularly in primary care, I know nurse practitioners have been wanting it for a long time.‘* (NP3)

*‘I think also it’s important to recognise that the standard of care can be maintained. So we’re getting really good outcomes, we’re providing convenience to everybody, both clinician and to patients, but the standard of care hasn’t been compromised, in my opinion, and if anything, I think it’s better.’* (NP7)

Participants also acknowledged that the rapid change meant that moving forward there is a need to review and reflect on the models to ensure that they are best practice:

*‘I think it’s all just a brave new world and everyone’s doing things a little bit on the fly, and when we maybe catch a chance to catch our breaths and look back and reflect properly, we might find things that we could do better.’* (GP1)

Existing models and expertise in other health contexts were seen to be a source of insight to inform further development of telehealth in mainstream PHC:

*‘Primary care needs to work through and work out how to do, so that’s sort of the uses of the tool really in different contexts. We could probably learn a lot from the guys that consult with people in Antarctica and things like that … I bet they’ve got a whole lot of techniques that we haven’t even thought of.‘* (GP11)

### Sustainability of telehealth

The following four subthemes impacted on the sustainability of PHC telehealth services: 1) the funding model; 2) maintaining patient and provider safety; 3) hybrid service models; and 4) access to support.

#### The funding model

The sustainability of telehealth services was seen as largely dependent on the funding model. Many participants described the importance for ongoing telehealth funding to be flexible and outcomes focused:

*‘… If we were logical about this, if we paid GPs for their outcomes, not their input, OK? So if you said to me, …“we are going to pay you for reducing those hospital admissions“ then I would use it intelligently. I would probably intersperse face to face with telehealth, I would do short catch-ups, I would do case conferences with me, the psychologist, the dietician and the patient with the eating disorder and together we would reduce the budget. It’s been shown around the world, we know how to do this.’* (GP12)

Nurses, in particular, noted that flexibility and fairness was required to recognise that different health professionals have varying types of consultations with patients and funding needs to reflect this difference:

*‘I think it’s a lot better for the doctors, a lot worse for AH, me included. Because we’re often doing the education, which takes a lot longer.’* (RN3)

*‘As a nurse practitioner we don’t get a — well, we don’t, it’s not particularly good, it’s actually just disgraceful, MBS rebate, so it makes it much more difficult for us to earn money than it does for doctors. So when people are not paying, it just makes it much, much harder again for us to cover costs.’* (NP5)

#### Maintaining patient and provider safety

Telehealth was considered by all participant groups to improve access to services and was a convenient alternative to face-to-face care that maintained the safety of patients and providers:

*‘So the fact that we could at least see some patients is a big positive.’* (AH clinician3)

*‘Well, it’s saved our life, and multiple others, basically ... without telehealth I don’t think that we would have been able to safely provide care for our patients for the past two years.‘* (GP1)

*‘We’re tending to do more, some of our chronic disease management over the phone just because it reduces the time that the patient and I are sitting in a confined space for an hour whilst we chat to them.‘* (RN5)

#### Hybrid service models

GP and RN participants broadly felt that a hybrid approach with a combination of face-to-face and telehealth consultations was a preferred ongoing model of service delivery:

*‘Telehealth should never be considered complete compared to face to face.‘* (GP7)

*‘No practice should be able to run where they are purely running telehealth.‘* (GP2)

*‘… there would be an initial face-to-face consultation, but after that, once the person is using their insulin and I’m confident that they understand the technical aspects of it, really all we’re doing is checking progress. So that can very effectively be done by telephone.’* (NP7)

Conversely, some AH clinicians indicated preference for face-to-face consultations where it was safe to do so, rather than a blended model of face to face and telehealth:

*‘My preference is absolutely face to face. Like I said, I’ve got five that are Zoom who travel a long distance. And it’s been quite good because it hasn’t upset things then for them when we’ve had lockdowns and whatever. The rest I just face to face.’* (AH clinician5)

The sustainability of telehealth will require a delivery model that meets the needs of different population groups. Many participants reported that their patients appreciated the convenience, flexibility, and increased access to services created by telehealth:

*‘I think they* [patients] *really like it … I think it’s been an eye-opener for a lot of patients ... it’s been quite odd and strange and new to start with, but most of them, most patients now quite like it.’* (GP3)

*‘... a lot of them are, “This is fantastic, I don’t have to come in. I can get my script, I can do what I want.“ So for those ones, it’s good*.*‘* (NP8)

While all participant groups spoke positively about the sustainability of telehealth post-pandemic, some participants identified some patient groups as preferring face-to-face consultations to telehealth. These included patients living with mental health conditions and older clients:

*‘... it was a mixture … when I first went back to face to face, it was about 50–50, people still wanted to go on with the video.’* (AH clinican4)

*‘So people were leaving their psychologist and coming to see me because their psychologist was only offering telephone or video telehealth … I think they voted with their feet — in any one week there might be 10% of my clients choose telehealth and 90% choose face to face.’* (NP2)

*‘I think a lot of our isolated elderly patients actually get benefit from physically coming in and chatting to our receptionist or our nurse or us rather than doing something remotely. I mean, half of the things we do for that group is social, not medical.’* (GP8)

#### Access to support

Several participants discussed the need for infrastructure, education, and practice standards to support and sustain future telehealth services. Examples of these included expanding telemonitoring services and developing professional standards to guide practice:

*‘... there are all kinds of interesting technological things, of course, so the more home monitoring devices people have, the better. So if we were wondering what their blood pressure was, they could measure it, and their temperature and their pulse and their oxygen level and their weight and their blood sugar and their cholesterol. You know, the more they could do at home, the less we need to do for them.’* (GP5)

*‘I think having some standards in place would be good around that as well to say, “Look, when you hit these certain points in your telehealth consult, consider that you might need to bring the patient in … perhaps having some standards in place would be helpful”.’* (NP3)

Improving public awareness about the use of telehealth and upskilling providers was perceived as important to enhance user confidence and to sustain the future viability of telehealth:

*‘I think coaching or training our patients to be prepared for telehealth in terms of both the interface, to start with, especially if they’re new to it, it takes, you lose a bit of time for people to just log in and getting all their credentials logged up and stuff, so there’s a bit of loss and wastage of time there.’* (GP10)

*‘I think it probably does need to be considered as a specific skill in terms of education and training. We were very lucky to have a couple of doctors who work quite extensively in rural telehealth services, which was really good when we first started doing telehealth, they could give us a lot of support and advice.‘* (GP9)

*‘I’m not sure what’s available ... probably I could do with a large amount of education. I haven’t really looked for or had time to look for it.’* (NP8)

## Discussion

### Summary

This article has reported on the lessons learnt by multidisciplinary PHC clinicians following the rapid introduction of telehealth in Australia owing to the COVID-19 pandemic, and their views on its future sustainability. Participants described the value of telehealth, its future potential, and how quality of care was perceived comparable with face-to-face visits with access benefits. The study demonstrated that telehealth could successfully be used by multidisciplinary PHC professionals in diverse settings. It has also suggested that funding models should be outcomes focused to remunerate appropriately for different types of consultation depending on patient needs. There was agreement that a hybrid approach, combining face to face and telehealth, would be preferable. Support for health professionals is required to sustain telehealth services, including training to address knowledge gaps and develop telehealth skills.

### Strengths and limitations

Exploring the use of telehealth in PHC from a multidisciplinary perspective was a key study strength; however, the small numbers of AH clinicians rendered it difficult to draw specific conclusions from the AH perspective. This study only explored the views of PHC providers and did not capture patients’ views. Further research is therefore required to identify benefits and challenges of telehealth expansion from the patients’ perspective. Furthermore, additional research is required to identify specific patient groups who benefit most from telehealth.^14^

### Comparison with existing literature

With the rapid switch to telehealth owing to the COVID-19 pandemic, several reports have confirmed the expanded role telehealth may play in the post-pandemic era.^2,14–16^ While Wright *et al* ^17^ endorsed the findings that individual resilience and flexibility were key factors in successful transitions, others described health professional resistance to telehealth owing to poor funding, inadequate training of health professionals, and lack of technological support.^18^

The present study’s finding that telephone was the preferred consultation method suggests that its familiarity, availability, and ease of use were likely factors, as has been previously reported.^2,8,16^ However, while telephone-based interventions have been found effective in supporting chronic condition management, video consultations improve diagnostic accuracy through visual capabilities.^8,19,20^ The study identified that a hybrid model combining telehealth with face-to-face consultations is preferable. Such a hybrid model would address potential limitations of telehealth, including safety concerns related to missed diagnoses. Future hybrid approaches have been described in several international studies.^5,21–23^ Jabbarpour *et al* ^22^ noted that out of 850 million doctor–patient encounters in the US pre-COVID-19, only 66% required face-to-face consultations, with the commonest reasons being for clinical ‘wellness’ reviews such as pap smears and immunisations. Findings such as these acknowledge that certain procedures and groups will continue to require face-to-face care interspersed with telehealth consultations.

Funding models were identified in the study as complex, fluid, and inflexible in not reflecting the different consultation types and durations. To this end, funding is a key potential barrier to the future sustainability of telehealth services. Participants in the study identified the need for telehealth funding to be available for both telephone and video consultations, whereas others have advocated that telehealth services provided by videoconferencing should receive increased funding owing to the potential to facilitate improved patient outcomes.^8^

One of the challenges associated with telehealth implementation in Australia has been the ongoing changes to service funding.^16,24^ Initially only limited funding was available for general practice nurses and more recently the Australian Government has announced the cessation of funding for long telephone consultations (>20 minutes) in July 2022.^16^ In addition, there is a proposal to reduce the extent to which telehealth is provided via telephone only in primary care in the future, with a preference for ongoing telehealth services to utilise videoconferencing.^25^ Changes such as these support the views of the study participants that the complexities of funding telehealth restrict its full potential, with the Royal Australian College of General Practitioners^26^ calling for simplification of telehealth funding rules.^27^ Several studies internationally^28–30^ and in Australia^1,16^ have identified issues with funding of telehealth services and the present study’s findings suggest that these issues need to be resolved to mainstream telehealth consultations into PHC models.

Supporting health professionals to become skilled in providing clinical care via telehealth was identified in the study as vital to ensure sustainability. This finding has been echoed by regulators^31^ and professional organisations.^32–34^ Areas for skill development include communicating effectively via digital platforms, conducting virtual assessments, identification of services safe to provide via telehealth or when face-to-face care is recommended, and issues relating to privacy.^1,3,4,6^ Similarly, international studies have called for practice standards and inclusion of telehealth education in undergraduate and postgraduate training programmes.^1,7,35–37^

### Implications for research and practice

The study findings provide evidence of factors likely to influence the sustainability of telehealth for PHC professionals. With further changes occurring in the funding of telehealth, the impact of these need to be investigated to ensure telehealth remains part of PHC delivery.

Further research to gain a better understanding of patients’ views of telehealth would add value to these findings and would ensure that health professionals are upskilled not just in the technological aspects of telehealth, but also in addressing specific needs and concerns of communities and individuals engaged in telehealth consultations, such as safety and privacy.^24^

In conclusion, the uptake of telehealth by PHC professionals during the COVID-19 pandemic has saved many lives. Two years down the track, lessons learnt from the COVID-19 pandemic in this study have highlighted key factors that may impact on the future sustainability of telehealth. By incorporating telehealth into a hybrid model of care delivery provided by well-trained and upskilled health providers, the delivery of PHC will better meet the needs of all in the community. Policymakers must pay attention to the concerns relating to reimbursements for telehealth provision to ensure multidisciplinary professionals continue to offer telehealth when most appropriate.

## References

[1] James S, Ashley C, Williams A (2021). Experiences of Australian primary healthcare nurses in using telehealth during COVID-19: a qualitative study. BMJ Open.

[bib2] Snoswell CL, Caffery LJ, Haydon HM (2020). Telehealth uptake in general practice as a result of the coronavirus (COVID-19) pandemic. Aust Health Rev.

[3] White SJ, Nguyen A, Roger P (2022). Experiences of telehealth in general practice in Australia: research protocol for a mixed-methods study. BJGP Open.

[4] Fisher K, Davey AR, Magin P (2022). Telehealth for Australian general practice: the present and the future. Aust J Gen Pract.

[5] Kichloo A, Albosta M, Dettloff K (2020). Telemedicine, the current COVID-19 pandemic and the future: a narrative review and perspectives moving forward in the USA. Fam Med Community Health.

[6] Royal Australian College of General Practitioners (RACGP) On demand telehealth services. https://www.racgp.org.au/advocacy/position-statements/view-all-position-statements/health-systems-and-environmental/on-demand-telehealth-services#ref-num-1.

[7] Odendaal WA, Anstey Watkins J, Leon N (2020). Health workers’ perceptions and experiences of using mHealth technologies to deliver primary healthcare services: a qualitative evidence synthesis. Cochrane Database Syst Rev.

[8] Thomas EE, Haydon HM, Mehrotra A (2022). Building on the momentum: sustaining telehealth beyond COVID-19. J Telemed Telecare.

[9] Jonnagaddala J, Godinho MA, Liaw S-T (2021). From telehealth to virtual primary care in Australia? A rapid scoping review. Int J Med Inform.

[10] Halcomb E, McInnes S, Williams A (2020). The experiences of primary healthcare nurses during the COVID-19 pandemic in Australia. J Nurs Scholarsh.

[11] Doyle L, McCabe C, Keogh B (2020). An overview of the qualitative descriptive design within nursing research. J Res Nurs.

[12] Halcomb EJ, Ashley C, Dennis S (2023). Telehealth use in australian primary healthcare during COVID-19: a cross-sectional descriptive survey. BMJ Open.

[13] Braun V, Clarke V, Cooper H, Camic PM, Long DL (2012). APA handbook of research methods in psychology, vol 2: research designs: quantitative, qualitative, neuropsychological, and biological.

[14] Imai C, Thomas J, Hardie R-A (2022). Telehealth use in patients with type 2 diabetes in Australian general practice during the COVID-19 pandemic: a retrospective cohort study. BJGP Open.

[bib15] Walia B, Shridhar A, Arasu P, Singh GK (2021). US physicians’ perspective on the sudden shift to telehealth: survey study. JMIR Hum Factors.

[16] De Guzman KR, Snoswell CL, Giles CM (2022). GP perceptions of telehealth services in Australia: a qualitative study. BJGP Open.

[17] Wright M, Versteeg R, Hall J (2020). General practice’s early response to the COVID-19 pandemic. Aust Health Rev.

[18] Kruse CS, Krowski N, Rodriguez B (2017). Telehealth and patient satisfaction: a systematic review and narrative analysis. BMJ Open.

[19] Rush KL, Howlett L, Munro A, Burton L (2018). Videoconference compared to telephone in healthcare delivery: a systematic review. Int J Med Inform.

[20] Gomez T, Anaya YB, Shih KJ, Tarn DM (2021). A qualitative study of primary care physicians’ experiences with telemedicine during COVID-19. J Am Board Fam Med.

[21] Knierim K, Palmer C, Kramer ES (2021). Lessons learned during COVID-19 that can move telehealth in primary care forward. J Am Board Fam Med.

[bib22] Jabbarpour Y, Jetty A, Westfall M, Westfall J (2021). Not telehealth: which primary care visits need in-person care?. J Am Board Fam Med.

[23] Johnson C, Dupuis JB, Goguen P, Grenier G (2021). Changes to telehealth practices in primary care in New Brunswick (Canada): a comparative study pre and during the COVID-19 pandemic. PLoS One.

[24] Willcock SM, Cartmill JA, Tse T (2022). How will telehealth change primary care in Australia?. BJGP Open.

[25] Australian Government Department of Health and Aged Care (2022). Continuing MBS telehealth services. http://www.mbsonline.gov.au/internet/mbsonline/publishing.nsf/Content/81F4D6E6C09A3762CA25887200043384/$File/Factsheet-telehealth-GPs-OMP.08.07.22.PDF.

[26] RACGP (2022). RACGP welcomes compliance delay but urges further action needed on telehealth. https://www.racgp.org.au/gp-news/media-releases/2022-media-releases-1/july-2022/racgp-welcomes-compliance-delay-but-urges-further.

[27] RACGP (2022). RACGP urges action on telehealth. https://www.racgp.org.au/gp-news/media-releases/2022-media-releases-1/june-2022/racgp-urges-action-on-telehealth.

[28] Chang JE, Lai AY, Gupta A (2021). Rapid transition to telehealth and the digital divide: implications for primary care access and equity in a post-COVID era. Milbank Q.

[bib29] Garcia R, Adelakun O (2017). A review of patient and provider satisfaction with telemedicine. http://www.robertjgarcia.com/AMCIS_2017_ER.pdf.

[30] Hamadi HY, Zhao M, Haley DR (2022). Medicare and telehealth: the impact of COVID-19 pandemic. J Eval Clin Pract.

[31] Australian Health Practitioner Regulation Agency (AHPRA) (2020). Telehealth guidance for practitioners. https://www.chiefpsychiatrist.wa.gov.au/wp-content/uploads/2020/06/Ahpra-FAQ-Telehealth-guidance-for-practitioners.pdf.

[32] RACGP (2022). Guide to providing telephone and video consultations in general practice. https://www.racgp.org.au/FSDEDEV/media/documents/Clinical%20Resources/Guidelines/Guide-to-providing-telephone-and-video-consultations.pdf.

[bib33] Australian Nursing Federation (ANF) (2013). Telehealth standards: registered nurses. https://www.anmf.org.au/media/lnfbx4r0/telehealth_standards_registered_nurses.pdf.

[34] Allied Health Professions Australia (2020). Telehealth guide for allied health professionals. https://ahpa.com.au/wp-content/uploads/2020/06/AHPA-Telehealth-Guide_Allied-Health-Professionals-May-2020.pdf.

[35] MacNeill V, Sanders C, Fitzpatrick R (2014). Experiences of front-line health professionals in the delivery of telehealth: a qualitative study. Br J Gen Pract.

[bib36] Kaplan B (2020). Revisiting health information technology ethical, legal, and social issues and evaluation: telehealth/telemedicine and COVID-19. Int J Med Inform.

[37] Rutledge C, Gustin TS (2021). Preparing nurses for roles in telehealth: now is the time!. Online J Issues Nurs.

